# Three-Dimensional Assemblies of Edge-Enriched WSe_2_ Nanoflowers for Selectively Detecting Ammonia or Nitrogen Dioxide

**DOI:** 10.1021/acsami.2c16299

**Published:** 2022-12-05

**Authors:** Aanchal Alagh, Fatima Ezahra Annanouch, Ayrton Sierra-Castillo, Emile Haye, Jean-François Colomer, Eduard Llobet

**Affiliations:** † Department d’Enginyeria Electronica, Universitat Rovira I Virgili, Avenida Paisos Catalans 26, 43007Tarragona, Spain; ‡ Laboratoire de Physique du Solide (LPS), Namur Institute of Structured Matter (NISM), University of Namur, Rue de Bruxelles, 61, 500Namur, Belgium; § Laboratoire d’Analyse par Réactions Nucléaires (LARN), Namur Institute of Structured Matter (NISM), Université de Namur, Rue de Bruxelles 61, 5000Namur, Belgium

**Keywords:** gas sensor, transition metal dichalcogenide, tungsten diselenide, chemical vapor deposition, nitrogen dioxide, ammonia

## Abstract

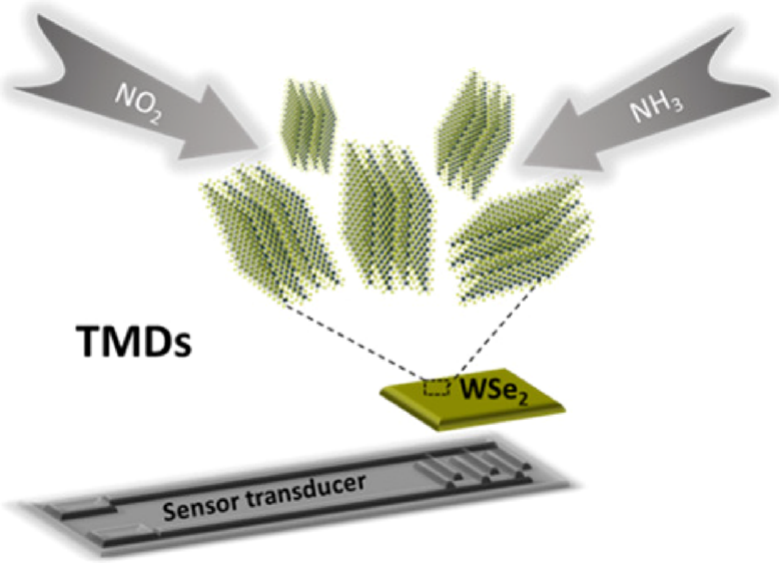

Herein, we present, for the first time, a chemoresistive-type gas sensor composed of two-dimensional WSe_2_, fabricated by a simple selenization of tungsten trioxide (WO_3_) nanowires at atmospheric pressure. The morphological, structural, and chemical composition investigation shows the growth of vertically oriented three-dimensional (3D) assemblies of edge-enriched WSe_2_ nanoplatelets arrayed in a nanoflower shape. The gas sensing properties of flowered nanoplatelets (2H-WSe_2_) are investigated thoroughly toward specific gases (NH_3_ and NO_2_) at different operating temperatures. The integration of 3D WSe_2_ with unique structural arrangements resulted in exceptional gas sensing characteristics with dual selectivity toward NH_3_ and NO_2_ gases. Selectivity can be tuned by selecting its operating temperature (150 °C for NH_3_ and 100 °C for NO_2_). For instance, the sensor has shown stable and reproducible responses (24.5%) toward 40 ppm NH_3_ vapor detection with an experimental LoD < 2 ppm at moderate temperatures. The gas detecting capabilities for CO, H_2_, C_6_H_6_, and NO_2_ were also investigated to better comprehend the selectivity of the nanoflower sensor. Sensors showed repeatable responses with high sensitivity to NO_2_ molecules at a substantially lower operating temperature (100 °C) (even at room temperature) and LoD < 0.1 ppm. However, the gas sensing properties reveal high selectivity toward NH_3_ gas at moderate operating temperatures. Moreover, the sensor demonstrated high resilience against ambient humidity (Rh = 50%), demonstrating its remarkable stability toward NH_3_ gas detection. Considering the detection of NO_2_ in a humid ambient atmosphere, there was a modest increase in the sensor response (5.5%). Furthermore, four-month long-term stability assessments were also taken toward NH_3_ gas detection, and sensors showed excellent response stability. Therefore, this study highlights the practical application of the 2H variant of WSe_2_ nanoflower gas sensors for NH_3_ vapor detection.

## 1 Introduction

Year by year, gas sensors have known tremendous developments in terms of sensing materials, size, power consumption, and fabrication costs. They are becoming indispensable items in the monitoring of indoor and outdoor toxic gases and thus play an increasing role in environmental monitoring, air quality control, or in safety and security applications.^1−4^ Among the main toxic and air pollutant gases, we find nitrogen dioxide (NO_2_) and ammonia (NH_3_), which are generated from motor vehicle exhaust, refineries, power plants, and so forth. While nitrogen dioxide has a direct contribution in the formation of ground-level ozone in the troposphere, acid rain and is a precursor of inorganic ambient particulate matter, ammonia contributes to acidification, eutrophication and is also a precursor for the formation of ammonium salts, a harmful form of fine particulate matter.^5−7^ For instance, exposure to nitrogen dioxide and ammonia may cause chronic bronchitis, respiratory irritation, mucous membrane inflammation syndrome, and asthma.^8,9^ The odor thresholds of NO_2_ and NH_3_ in the air are around 400 and 40 ppb, respectively, while the threshold limit values (TLV) have been set by the Occupational Safety and Health Administration (OSHA) to 5 and 25 ppm, respectively.^10^ Exposure to high concentrations of these gases is a serious health threat. Hence, developing a new generation of gas sensors that can monitor such pollutant gases in real time and can detect concentrations from as low as a few parts per billion to several hundred parts per million in the air is of strong industry demand. In this context, chemiresistive sensors based on nanostructured metal oxide semiconductors, such as WO_3_, ZnO, SnO_2_, and In_2_O_3_, only to cite a few^5,11−13^ have been extensively used for detecting pollutant gases, owing to their advantages of sensitivity, low cost, simple fabrication process, and reliability. They were launched as building block materials for gas sensors, owing to their high surface-to- volume ratio, low number of defects, electron confinement effect, and so forth.^14−16^ However, their lack of selectivity and humidity cross-sensitivity remain the major drawbacks to overcome. Additionally, these nanomaterials are normally operated at 100–400 °C, leading to high power consumption and reduced sensor stability and lifetime, owing to thermally induced changes in morphology and poisoning effects. These drawbacks limit the adoption of metal oxide nanomaterial chemiresistors in wider real-time applications.

In a quest for overcoming such drawbacks, researchers have recently drawn toward atomically layered two-dimensional (2D) transition metal dichalcogenide (TMD) nanomaterials. TMDs possess unique properties such as semiconducting properties, direct band gaps, and high specific surface areas because of their sheet-like structures with large basal planes and highly reactive edges.^17,18^ TMDs consist of a metal atomic layer (such as Mo, W, Hf, Ti, Zr, V, Nb, Ta, Re, etc.) collocated between two chalcogen atomic layers (S, Se, or Te), and then, these 2D trilayers may appear stacked in multilayer structures because of van der Waals interactions.^19^ Among the TMD nanomaterials that have proven their feasibility as a gas-sensing element, we cite tungsten diselenide (WSe_2_), which has ultimately sparked the interest of many researchers, especially in the detection of NO_2_ and ammonia. Guo and co-workers^20^ or Zhang and co-workers^21^ have reported ultrasensitive room temperature NO_2_ sensors based on liquid-phase exfoliated WSe_2_ nanosheets. Medina and co-workers^22^ synthesized wafer-scale WSe_2_ monolayers toward phase-engineered hybrid WOx/WSe_2_ films with sub-ppb NOx gas sensing by a low-temperature plasma-assisted selenization process. Ko and co-workers^23^ developed high-performance NO_2_ and NH_3_ gas sensors based on three-layer WS_2_ nanomaterials. Up to now, most of the reported studies have synthesized WSe_2_ in a two-dimensional direction, in the form of mono or multilayer nanosheets. However, it has been reported that a vertical orientation (3D) of these nanomaterials is highly advantageous for gas sensing applications.^24−26^ Such an arrangement offers a large surface area to volume ratio with an enriched number of exposed edge sites and an increased number of defects that make the nanomaterial highly reactive with gas molecules.^27^ In addition, the 3D arrangement of TMD nanosheets offers plenty of voids for gas diffusion. Indeed, gas adsorption at the edge sites of TMDs is more important compared to their basal plane, which has minimal dangling bonds, and, with the effect of thermodynamic forces, it is very challenging to expose the edges of 2D TMDs to the environment when these lie flat on the application substrate.^7,17,28,29^

In this respect, there are two techniques that are mostly used for the growth of 3D assemblies of edge-enriched WSe_2_ nanosheets: hydrothermal and atmospheric pressure chemical vapor deposition methods.^30−32^ Hydrothermal synthesis has the advantage of being low cost; however, it is a time-consuming technique (it involves multistep fabrication), and sometimes, it needs using hazardous precursors, which have hindered its development. In contrast, atmospheric pressure chemical vapor deposition (CVD) results in the synthesis of multi-layered TMDs with high yield, while enabling the control in the number of layers. Moreover, it is a catalyst-free technique, and it allows the direct growth of the material on the application substrate, that is, the alumina sensor transducer.

Herein, we report, for the first time, on the successful synthesis of 3D assemblies of edge-enriched tungsten diselenide nanoflowers, using a combination of aerosol-assisted CVD and atmospheric pressure CVD techniques, for the development of bifunctional NO_2_ and NH_3_ gas sensors. The films were directly synthesized on alumina substrates, at atmospheric pressure without the need of any catalyst seeding. The grown films were studied in detail by using scanning electron microscopy (SEM), field emission SEM (FESEM), and high-resolution transmission electron microscopy (HRTEM) to analyze their morphology; X-ray diffraction (XRD) to determine their structure; and Raman and X-ray photoelectron spectroscopy (XPS) to define their composition. Additionally, the films were studied against small concentrations of NO_2_ and NH_3_ at low operating temperatures ranging from room temperature (i.e., 25 °C) to 150 °C. Besides, sensor performance was examined in the presence of ambient moisture. It is worth noting that only one previous paper^24^ has reported so far the synthesis and use of 3D assemblies of WSe_2_ nanoflowers for gas sensing. Finally, a comparison study between our work and the previous literature is reported and the gas sensing mechanism for both gases is introduced and discussed.

## 2 Experimental Details

### 2.1 Material Synthesis

3D assemblies of edge-enriched WS_2_ nanoplatelets were synthesized in two steps. First, WO_3_ nanowires were grown directly onto commercial alumina substrates (Ceram Tech GmBH, Germany) by using aerosol-assisted CVD method (AACVD). More details can be found in our previous reports.^17,33^ The front side of these substrates comprises screen-printed interdigitated platinum electrodes (electrode gap of 300 μm), while the backside comprises a screen-printed Pt heater (having a resistance of 8 Ω) to enable setting the operating temperature of the sensor. In the second step, the as-grown nanowires were subjected to a double selenization process via an ambient-pressure CVD technique to achieve a tungsten diselenide nanomaterial. The synthesis process is adopted from our previous research work.^17^ As in a typical selenization process, two boats containing the Se powder (purity 99%) were placed into two temperature zones, one boat at 40 °C and the other at 850 °C (in total 700 mg), along with the WO_3_ nanofilm sample, which was placed at 850 °C prior to the selenization process, the quartz tube was flushed with a 0.475 L min^–1^ argon flow for 1 h, in order to remove oxygen. Next, 0.150 L min^–1^ of hydrogen (H_2_) flow was added to the argon flow. In the first step of 30 min, the Se powder was placed at the 850 °C zone. The optimized second selenization step was performed by inserting the quartz tube in the hot zone of the furnace such that the Se powder placed at 40 °C reaches a 400 °C temperature zone. The sample remained at 850 °C as the quartz reactor was moved over a few centimeters. After the reaction, the H_2_ flow was stopped, and the quartz tube was removed from the reactor (quartz tube) and was cooled with the argon flow for 1 h.

### 2.2 Characterization Techniques

The morphology of the as-grown WO_3_ nanowires was examined using a scanning electron microscope (JEOL 7500F microscope operating at 15 kV), whereas after the selenization of WO_3_ nanowires, the as-grown WSe_2_ nanoplatelets were analyzed using a field-emission scanning electron microscope Hitachi 2000 and FEI Helios Nanolab 650. TEM studies were carried out on a TECNAI T20 microscope working under 200 kV. To prepare a sample, the material was scratched from the surface of an alumina substrate, dispersed in ethanol using sonication, and a droplet was put on a holey-carbon copper grid. For chemical phase analysis, XRD measurements were made using a Bruker-AXS D8-Discover diffractometer. The chemical composition of the WO_3_ nanowires and WSe_2_ nanoplatelets was studied by XPS using an Escalab 250i Thermo Fisher spectrometer (Al Kα 1486.68 eV). The O 1s, Se 3d, and W 4f core levels have been recorded at a pass energy of 20 eV, with 20 scans, on a spot size of 250 × 250 μm. A flood gun has been used for charge compensation and no additional energy shifting is applied to the spectra. The authors are aware of the recent warnings about XPS analysis on insulating samples.^34−36^ The spectra are analyzed with Thermo Avantage software, considering a Shirley background. The Raman spectra were obtained using a Renishaw in Via, laser 514 nm, ion argon-Novatech, 25 mW.

#### 2.2.1 Gas Sensing Device Fabrication and Gas Sensing Measurements

**Figure 1:**
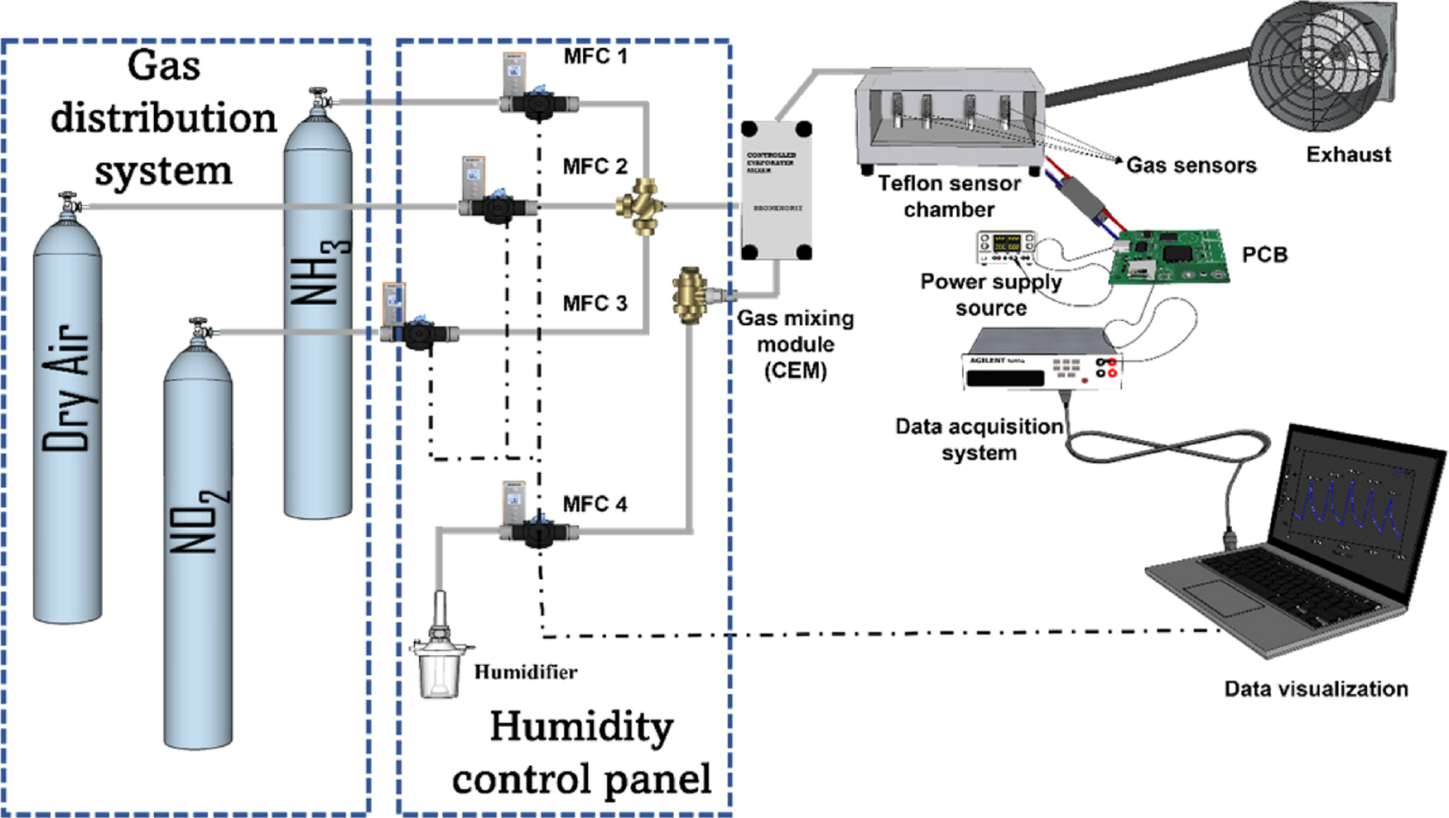
Schematic illustrating the home-made gas mixture, delivery, and sensor data acquisition systems.

The gas sensing characteristics of the fabricated WSe_2_ nanoflower sensors were measured by using a homemade gas-sensing detection system. A schematic diagram of the home-made gas sensing detection system is shown in Figure 1. The as-fabricated sensor was placed inside a Teflon test chamber of 35 mL in volume. This testing chamber was connected to a fully automated, continuous gas flow measurement set-up able to supply diluted gas mixtures as well as humidified gas mixtures using a mass flow controller (Bronkhorst High-Tech B.V.) and electro valves. The gases employed for testing were used from calibrated gas cylinders balanced in dry synthetic air (Air Premier purity: 99.999%). The operating temperature of the sensor was controlled by connecting its heater to an external power supply (Agilent, model 3492A).

The sensing measurements were done by recording the change in electrical resistance of the sensor upon exposure to several different concentrations of target gases such as NO_2_, NH_3_, H_2_, CO, and C_6_H_6_ at different operating temperatures (room temperature, 100 and 150 °C). A sensor was kept in a dry airflow for a period of 2 h before performing gas sensing measurements to stabilize its baseline resistance. Then, the sensor was exposed to a given concentration of a gaseous species for 10 min followed by 50 min exposure to dry air to regain and stabilize its baseline. The electrical resistance of the sensor was measured by using an Agilent-34972A multimeter. The gas flow and humidity were controlled using mass-flow controllers (MFC). Throughout the tests, the overall flow rate was maintained at 100 mL/min. To evaluate humidity interference, certain tests were done in a humid environment (e.g., 50% RH at 25 °C). While the sensor was exposed to varying concentrations of NO_2_ and NH_3_, the humidity level was kept constant. For an oxidizing gas, such as NO_2_, the sensor relative response was calculated by using eq 1, while in the case of a reducing gas (such as NH_3_), the gas sensing relative response value was calculated using eq 2. Even though the relative response, as defined in eqs 1 and 2, has been used throughout this paper, from now on, the term response is used for short.
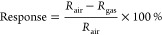
1
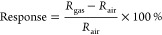
2Here, *R*_air_ and *R*_gas_ are, respectively, the real-time resistances of the sensor exposed to air and to a target gas. For practical applications, the response and recovery times are very important parameters. In this work, the response time of the sensor (*T*_res_) is defined as the time taken by the sensor to reach 90% of the total resistance change when exposed to a target gas, and the recovery time (*T*_rec_) is defined as the time required to recover from 90 to 10% of the total resistance change when the analyte is removed from the air flow.

## 3 Results and Discussion

### 3.1 Material Characterization

The morphological and chemical characteristics of the as-fabricated tungsten diselenide nanomaterial were assessed through various techniques, discussed in detail in this section.

#### 3.1.1 SEM and FESEM Characterization

**Figure 2:**
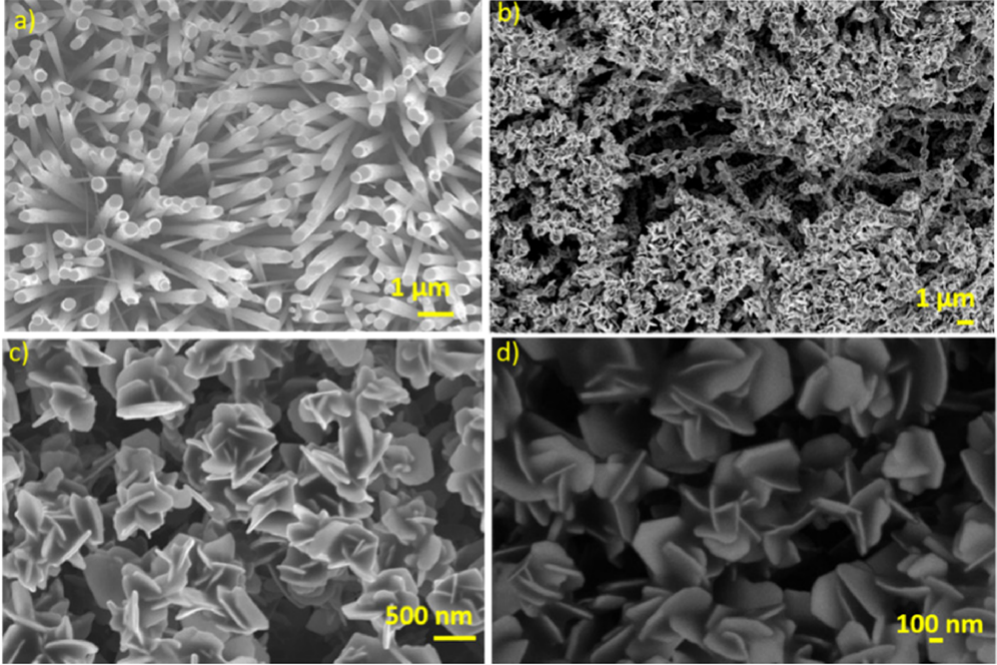
SEM and FESEM images depicting the growth of vertically aligned (a) WO_3_ nanowires, (b) WSe_2_ nanoplatelets, and (c,d) WSe_2_ nanoflowers.

The AACVD method was employed to grow adherent uniform films of tungsten trioxide nanowires on alumina substrates. A scanning electron microscope was used to analyze the morphology and microstructure of these nanowires, as presented in Figure 2a. The result demonstrates that the sensor substrate is homogeneously covered with thin nanowires of WO_3_. Even when showing different tilt angles, nanowires show a tendency to grow with vertical orientation to the substrate. The length of as-grown nanowires is in the range of 6–7 μm because these films are obtained using the same procedure as reported in our previous studies.^17,37^ Afterward, the as-grown WO_3_ films were selenized to produce WSe_2_ nanofilms, which were subsequently examined using a high-resolution scanning electron microscope (Figure 2b–d). It is evident from the results obtained that after undergoing the selenization process, the morphology of these nanowires changes completely, resulting in platelets with well-defined shapes and sharp edges. Moreover, the layer-stacked bulk platelets grow vertically, similarly to WO_3_ nanowires, as illustrated in Figure 2b. The results highlight that these platelets are evenly distributed and piled on top of one another, resulting in WSe_2_ nanowires with platelet attachments covering the entire length of the nanowires, as seen in Figure 2b. Furthermore, SEM pictures reveal nanoplatelets assembled in the form of nanoflowers at the tips of these nanowires (Figure 2c). Also, the higher magnification image presented in Figure 2d reveals the thickness of these platelets, which is in the range of 40–50 nm.

#### 3.1.2 TEM Characterization

**Figure 3:**
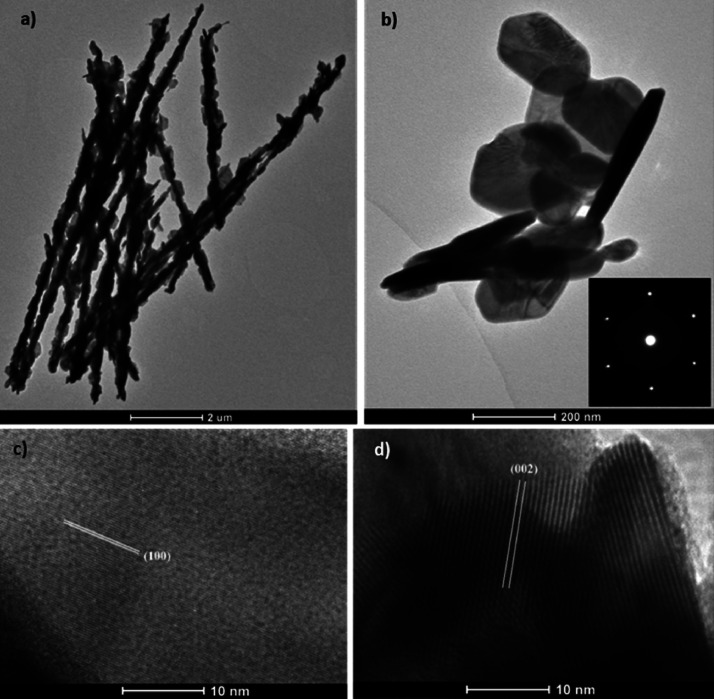
Low-magnification TEM images of (a) WSe_2_ nanowires and (b) WSe_2_ nanoflower petals with an inset of the SAED pattern along the [100] axis and high-resolution images of (c) a petal showing the (100) crystal planes and (d) layered structures of WSe_2_ nanoplatelets composed of the petals with the (002) interlayer spacing.

The morphology, structure, and crystallinity of the as-prepared nanoflowered material were further investigated using TEM (Figure 3). It can be observed in Figure 3a that WO_3_ nanowires completely transform into WSe_2_ nanowires after undergoing the selenization process. These WSe_2_ nanowires, which are about 10 μm in length, contain WSe_2_ nanoplatelets along their entire length. These nanoplatelets arrange themselves in nanoflowers at the tips of the nanoneedles, as shown in SEM, but not in Figure 3a, certainly due to the TEM preparation. However, these nanoplatelets composing the petals of these nanoflowers are found dispersed on the grid, as shown in Figure 3b. In the inset of Figure 3b, the selected area electron diffraction (SAED) pattern of a single petal was acquired along the [100] axis and reveals only a single set of diffraction points arranged in a hexagonal symmetry, demonstrating its high crystallinity. The (001) planes in Figure 3c have a lattice spacing of about 0.29 nm, which is consistent with the 2H-phase of WSe_2_. Furthermore, Figure 3d displays an interlayer spacing of roughly 0.66 nm, corresponding to the (002) plane of the hexagonal WSe_2_.

#### 3.1.3 Raman Characterization

**Figure 4:**
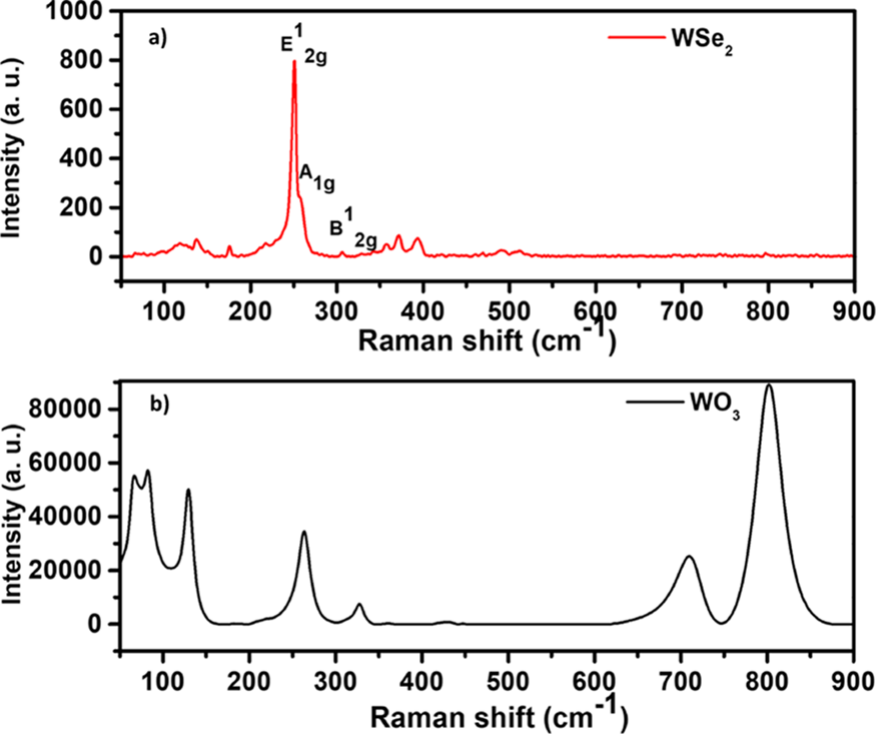
Raman spectra of (a) as-grown WSe_2_ nanoflowered film and (b) WO_3_ nanowires.

Raman spectroscopy is a powerful and nondestructive tool for assessing nanomaterial quality and device feasibility. It is also very useful to determine the number of layers that contribute to the film thickness of a sample. In this respect, Raman spectroscopy was used to further examine the grown samples. To check for the presence of tungsten oxide in the selenized samples, the Raman spectra of the starting material (WO_3_ nanowires) were compared to the films formed after the selenization process. Two spectra were obtained using a laser with an excitation wavelength of 532 nm, where the upper spectrum (Figure 4a) corresponds to the nanoflower film obtained after selenization of WO_3_ nanowires and the lower spectrum corresponds to the WO_3_ nanowires (Figure 4b). As presented in Figure 4a, two main peaks were obtained, the peak at 251 cm^–1^ corresponds to the E^1^_2g_ (in-plane vibration of the Se and W atoms) while a small shoulder peak at 257.6 cm^–1^ corresponds to the A_1g_ (out-of-plane vibration) modes. These are the two characteristic peaks associated with the presence of 2H-phase WSe_2_. An additional peak was observed at 306 cm^–1^ (B_2g_^1^ mode) which has been linked to interlayer interactions. The absence of this peak is associated with the growth of monolayers, which is not the case here.^37,38^ The Raman analysis results are consistent with prior publications, demonstrating that the as-grown WSe_2_ nanoflowers films are multilayered structures.^39,40^ Moreover, there was no other peak that indicated the presence of WO_3_ impurities or any other impurity. Furthermore, for comparison purposes, the Raman spectrum of WO_3_ NWs is shown in Figure 4b. All of the peaks (271, 327, 715, and 805 cm^–1^) are indicative of the monoclinic tungsten trioxide phase, which is consistent with our previous reported studies.^33,41^

#### 3.1.4 XRD Analysis

**Figure 5:**
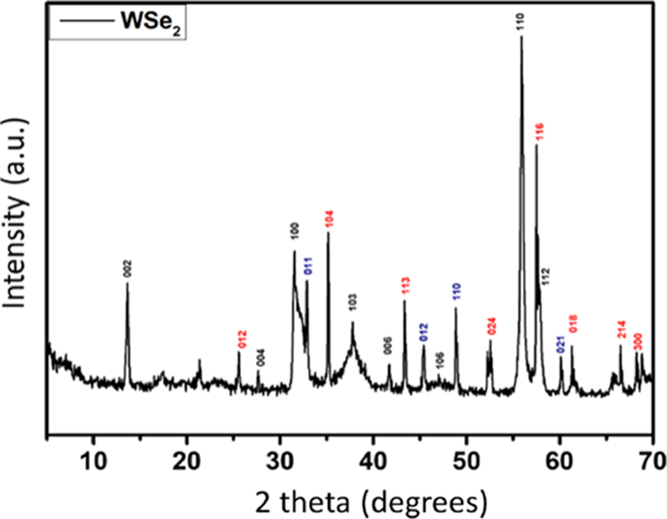
XRD diffractogram of as-grown WSe_2_ nanoflowered film on alumina substrates with screen-printed platinum electrodes. Peaks whose crystal planes appear in red correspond to the alumina substrate on which the WSe_2_ films were grown.

The crystallographic structure and the purity of the WSe_2_ nanoflower films are evaluated by the XRD technique. The XRD pattern obtained (Figure 5) reveals the presence of intense peaks at 13.65°, 27.50°, 31.44°, 37.86°, 41.78°, 47.43°, 55.99°, and 57.92°, in agreement with the (002), (004), (100), (103), (006), (105), (110), (112) crystal planes of the hexagonal WSe_2_. All the diffraction peaks are indexed to the ICDD card no 01-071-0600, confirming the presence of 2H phase WSe_2_, marked with black color in the diffractogram. The high-intensity peaks imply a highly crystalline material, having a crystallite size above 400 nm, also reported earlier.^42^ Additionally, peaks corresponding to the alumina substrate were also detected and their lattice planes are highlighted in red. It was found that the peaks at 32.9°, 45.3°, 49.0°, and 60.3° can be indexed to the (011), (012), (110), (021) crystal planes, indicating the presence of small amounts of platinum selenide sulfide, Pt(SSe). These diffraction peaks are colored blue in the spectra and are referenced to the ICDD card no. 01-078-9794. The platinum is from the interdigitated electrodes screen-printed on the alumina substrate. Moreover, the absence of diffraction peaks attributable to a different phase than the 2H confirms that single crystalline 2H-WSe_2_ is grown.

#### 3.1.5 XPS Analysis

**Figure 6:**
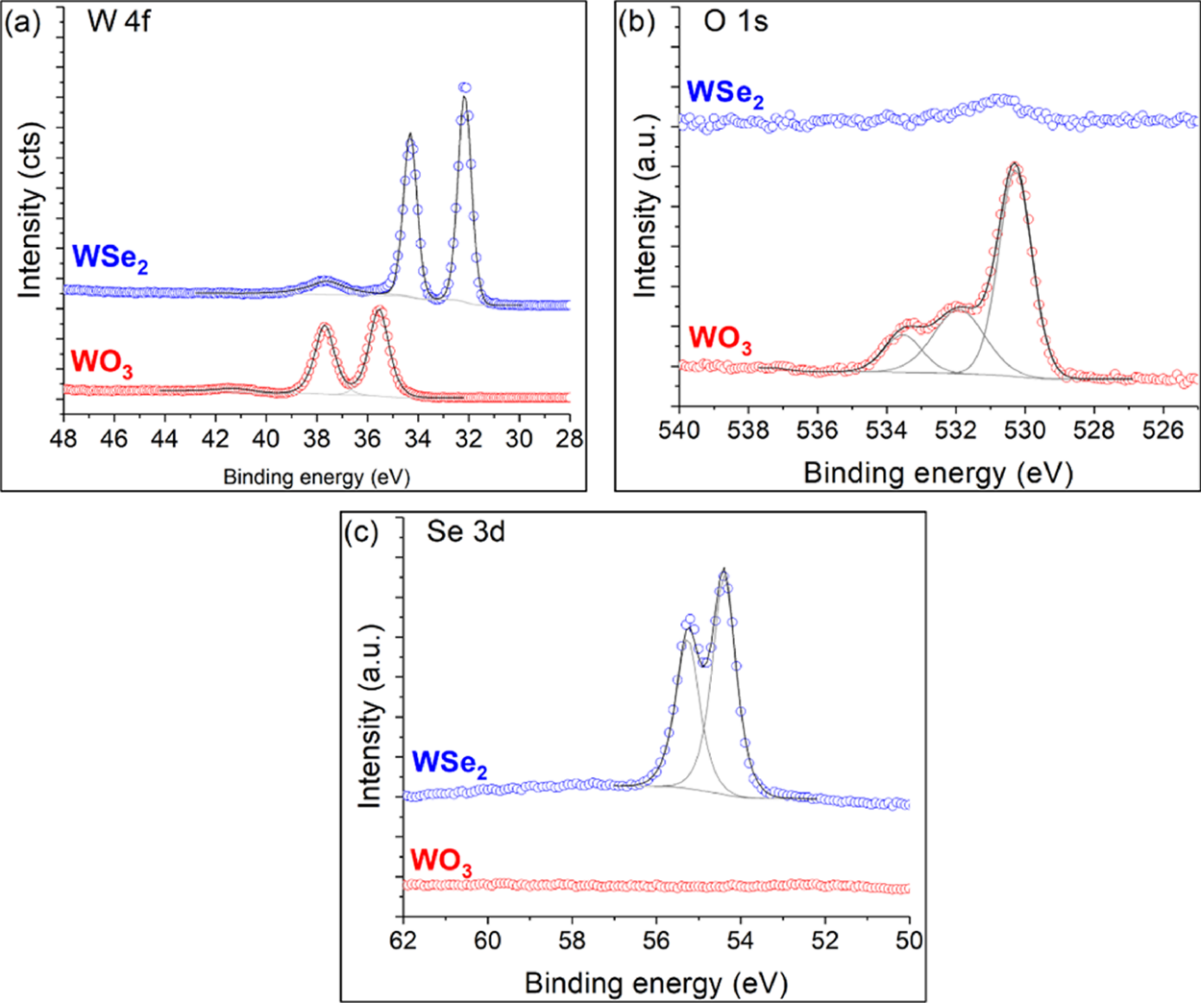
(a) W 4f, (b) O 1s, and (c) Se 3d XPS core-level spectra of WO_3_ nanowires and WSe_2_ nanoflowers.

To further investigate the chemical composition of the as-prepared material, an XPS analysis was performed. The analysis of the sample before and after selenization is shown in Figure 6, confirming that the tungsten oxide nanomaterial has been completely transformed. Before selenization, the W4f signal (Figure 6a) is composed of a doublet centered at 35.5 and 37.7 eV, corresponding to W 4f7/2 and W4 f5/2 and an additional contribution at 41.2 eV, attributed to the W 5p3/2 level. These positions are in agreement with WO_3_ formation.^43^ On the O 1s level (Figure 5b), an intense contribution at 530.3 eV, corresponding to WO_3_, confirms the oxide production. Two additional peaks are observed at 531.9 and 533.5 eV, attributed to organic oxygen (C–O, C=O), which are most likely from synthesis. As expected, no selenium is present before the selenization steps (Figure 6c). However, after selenization, the W 4f signal shifts to a lower binding energy and exhibits a unique doublet contribution centered at 32.2 and 34.3 eV, which is consistent with the WSe_2_ 2H phase formation, where the literature reports binding energies in the range 32.0 to 32.4 eV^44,45^ and up to 32.7 eV^46^ for large, oxygen-free tungsten diselenide flakes. Following the WSe_2_ 2H phase formation, the Se 3d signal is composed of a unique doublet contribution centered at 54.4 and 55.26 eV because of the Se 3d5/2 and Se 3d3/2 levels, respectively. After selenization, there is no evidence of significant oxygen presence. As a result, the presence of the 2H phase is confirmed by XPS, XRD, and Raman analyses, as the 1 T (or 1 T′) phase exhibits W 4f and Se 3d signals that are slightly shifted to lower binding energies (≈31.9 eV for the W 4f signal and 53.6–54.1 eV for Se 3d).^42,44,45^

In summary, the results of SEM and TEM demonstrate the growth of vertically aligned WSe_2_ nanoflowers. Furthermore, the results from the XRD analysis reveal that the as-grown WSe_2_ nanoflowers exhibit excellent crystallinity. The lack of diffraction peaks that can be associated to other phases also suggests that WSe_2_ nanoflakes are single crystalline. We may also conclude that the WSe_2_ nanoflowers have a multilayered structure based on the Raman and XRD findings.

#### 3.1.6 Gas Sensing Results

##### 3.1.6.1 NO_2_ Detection

The gas sensing properties of the as-fabricated WSe_2_ nanoflower sensors were evaluated using a homemade gas monitoring system (described in detail in the previous section). Choosing the optimal working temperature for a gas sensor is crucial for evaluating its performance because the sensitivity, selectivity, and response/recovery speed of gas-sensitive materials are all heavily dependent on the operating temperature. In order to identify a suitable operating temperature for detecting NO_2_, the temperature-dependent responses of the sensors to NO_2_ were initially investigated. For doing so, we subjected our sensors to 800 ppb NO_2_ in dry air balance and measured the resulting responses at various operating temperatures below 150 °C. Measuring a single gas concentration is a straightforward process for identifying an operating temperature that enhances response (and the signal-to-noise ratio). Setting 150 °C as the maximum operating temperature to be tested is due to the thermal instability of 2H-WSe_2_ at higher temperatures as well as to prevent ambient oxidation of 2H-WSe_2_. Investigating moderate operation temperatures is also beneficial for developing low-power devices.^17^

**Figure 7:**
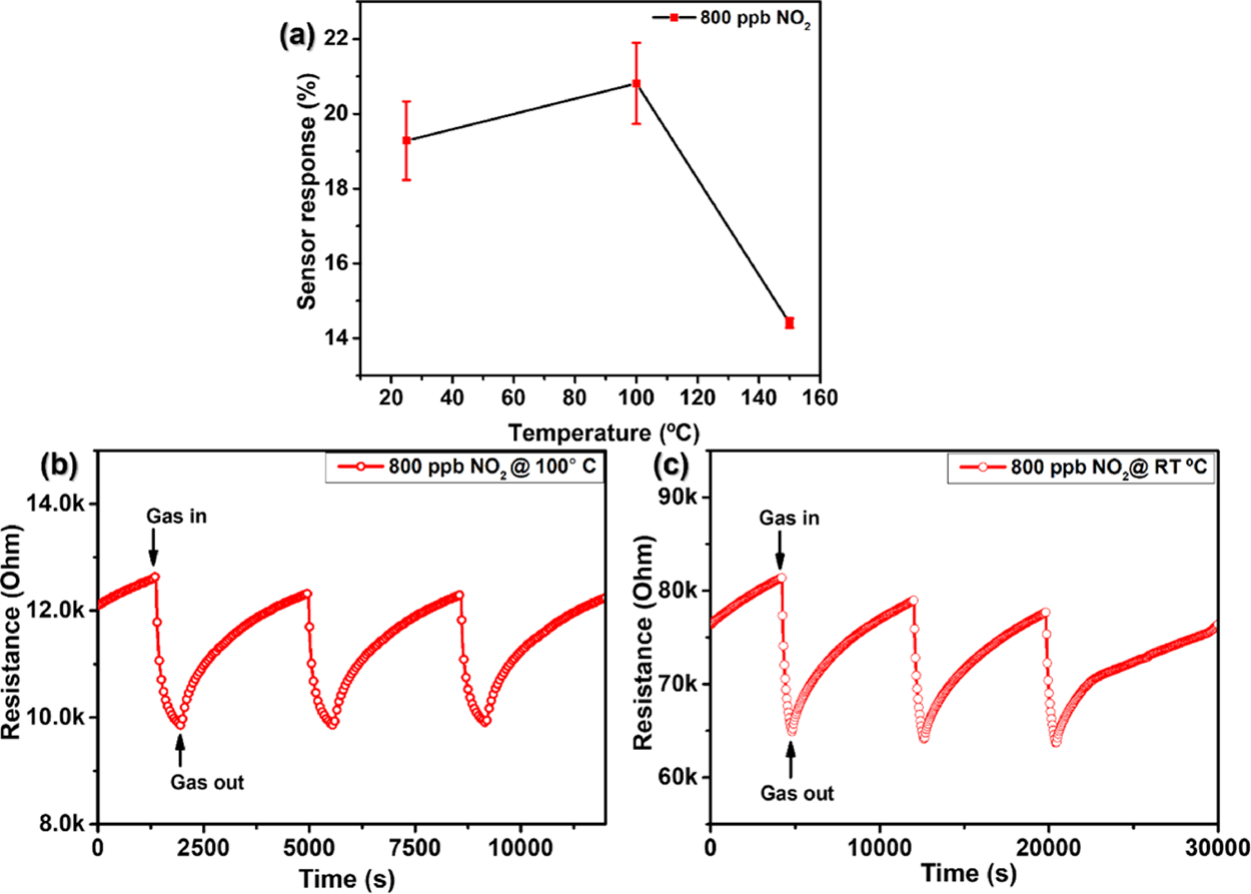
(a) Typical WSe_2_ sensor response as a function of temperature toward NO_2_ gas, (b) film resistance change as a function of time, toward 800 ppb of NO_2_ at 100 °C, and (c) film resistance change as a function of time toward 800 ppb of NO_2_ at 25 °C.

The sensor response to 800 ppb NO_2_ gas is shown in Figure 7a at varying operating temperatures ranging from 25 to 150 °C. As demonstrated, the sensor response increases with an increase in operating temperature from 25 to 100 °C, and then, it decreases as the temperature is increased to 150 °C. The presence of potential selenium vacancies is attributed for the increased response at 100 °C. For instance, it has been reported in the literature that these vacancies promote NO_2_ molecule adsorption at the WSe_2_ surface, thereby increasing the interaction with the target gas molecules.^47^

Furthermore, the sensor response is calculated to be 18.5% at room temperature (25 °C), rising to more than 20.5% at an operating temperature of 100 °C before dropping to only 14.2% at 150 °C. Consequently, 100 °C has been determined to be the optimal operating temperature for the fabricated WSe_2_ nanoflowered sensors for NO_2_ gas detection. It is worthy to mention that when compared to various metal oxides and other TMD materials, this operating temperature is relatively low.^48,49^ The response curve of gas sensors as a function of their operating temperature often is bell-shaped. The adsorption of gas molecules at the active sites of the gas-sensitive nanomaterial, eventual reaction with surface species, and associated charge transfer are thermally activated processes. However, too high an operating temperature promotes the desorption of gas molecules or limits the diffusion depth of gas molecules into a porous gas-sensitive film, thus reducing response intensity.^50^

Figure 7b,c shows an example of dynamic film resistance change in response to 800 ppb NO_2_ gas, at 100 and 25 °C, respectively. When exposed to an oxidizing gas, such as NO_2_, the WSe_2_ sensor responds as a p-type semiconductor with decreasing resistance. The electron–acceptor characteristics of oxidizing gases like NO_2_ can elucidate this behavior. When a p-type material is exposed to an oxidizing atmosphere, electrons are removed from the conduction band, increasing the hole density and decreasing the material’s electrical resistance and vice versa when exposed to a reducing atmosphere.^17^ Besides, it is observed that the sensor does not completely return to its baseline resistance when the target gas is withdrawn and the sensor is exposed to dry air; however, the baseline resistance is recovered when the temperature is raised to 100 °C. This is due to the fact that higher temperature promotes faster desorption of gas molecules, resulting in a faster recovery cycle. In addition, the sensor response and recovery time for 800 ppb NO_2_ varied considerably with temperature. For instance, at room temperature, the response and recovery times toward 800 ppb of NO_2_ gas are computed to be 411 and 5446 s (Figure S1a), respectively, while at 100 °C, they fall to 196 and 2218 s (Figure S1b). This decrease in response, as well as recovery times, is attributed to the much faster desorption of NO_2_ gas molecules at an elevated temperature.

**Figure 8:**
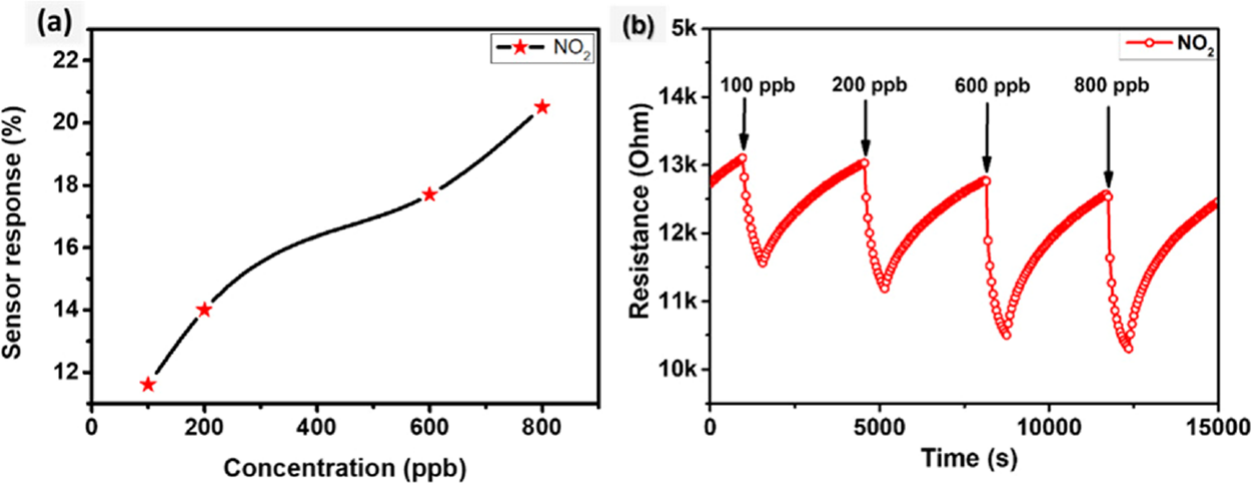
(a) WSe_2_ sensor response as a function of different NO_2_ concentrations and (b) film resistance change as a function of time, toward different NO_2_ concentrations, at 100 °C.

Furthermore, at an operating temperature of 100 °C, the sensors were tested against a wide range of NO_2_ gas concentrations ranging from 0.1 to 0.8 ppm. As expected, increasing NO_2_ concentrations leads to higher resistance changes, resulting in an enlarged sensing response. Figure 8 depicts the observed data, which indicate a superlinear increase in sensing response with each increase in gas concentration (Figure 8a).

An example of the dynamic film resistance change over time in response to various NO_2_ gas concentrations is presented in Figure 8b. The responses toward 0.1, 0.2, 0.6, and 0.8 ppm NO_2_ were calculated to be 11.5, 14, 17.6, and 20.4%, respectively. It is worth mentioning that the sensor demonstrated remarkable sensitivity and high stability when detecting lower concentrations. The WSe_2_ sensors, as shown in Figure 8b, can detect NO_2_ gas concentrations as low as 0.1 ppm, which is substantially lower than the threshold exposure limit of NO_2_ as recommended by the American Conference of Government Industrial Hygienists.^47^ Henceforth, it can be deduced that the sensor exhibited an exceptionally low LoD which is below 100 ppb at a moderate operating temperature (100 °C).

Moreover, Table 1 gives more insights by comparing the performance in the detection of NO_2_ reported in this paper with those found in the literature. The data reported in this table are comparable because unless clearly specified, sensors have been operated under similar conditions and the definitions of response, limit of detection, and response time are consistent throughout the references cited. From the results obtained, the fabricated WSe_2_ sensors show outstanding gas responses with high sensitivity toward NO_2_ detection and with LoD < 0.1 ppm when operated at 100 °C. Besides that, sensors remain functional and demonstrate acceptable sensitivity toward the targeted gas (NO_2_) even when operated at room temperature.

**Table 1:** Comparison of the NO_2_, NO, and NOx Sensing Performance of WSe_2_ Nanomaterials with Other Sensing Materials^a^

2D material	studied conc.	working temp. (°C)	response (%)	LoD (ppm)	gas detected	humidity cross-sensitivity test	response time	ref
**WSe_2_ NFs**	**0.8 ppm**	**100**	**20.5 ± 1.44**	**0.1 (exper.)**	**NO_2_**	**response increases slightly under wet conditions**	**196 s**	**this work**
WSe_2_ vertical ns	1 ppm	RT	34.6	0.04 (theor.)	NO_2_	response decreases with increase in R.H.	66 s	(24)
WSe_2_	2 ppm	RT	18.8	not available	NO_2_	not studied	not available	(56)
WSe_2_ vertical structure	500 ppm	RT +1 V	4.5	not available	NO	not studied	not available	(57)
hybrid WOx/WSe_2_	25 ppm	RT	13	0.3 (theor.)	NOx	not studied	250 s	(22)
WSe_2_ nanoscrews	1 ppm	RT	350	0.072 (theor.)	NOx	not studied	120 s	(58)
WS_2_ nanosheets	5 ppm	160	121	0.2 (exper.)	NO_2_	decrease in sensor response under wet conditions	not available	(49)
WS_2_ aerogel	3 ppm	250	0.36 (Δ*R*/*R*_0_)	0.008 (theor.)	NO_2_	decrease in sensor response under wet conditions	120 s	(59)
MoS_2_/graphene	5 ppm	150	1.08/5 ppm	1.2 (exper.)	NO_2_	not studied	not available	(60)
MoS_2_/rGO hybrid	3 ppm	160	1.29 *R*_a_/*R*_g_	0.1 (exper.)	NO_2_	high humidity influence on sensing response	8 s	(61)
WS_2_ graphene aerogel	2 ppm	180	3%	0.01 ppb (theor.)	NO_2_	sensor response increases under wet conditions	100 s	(62)

a NFs: nanoflowers; 5 L: 5 layers; ns: nanosheets; conc.: concentration; exper.: experimentally verified; theor.: theoretically calculated; LoD: limit of detection.

##### 3.1.6.2 NH_3_ Sensing

**Figure 9:**
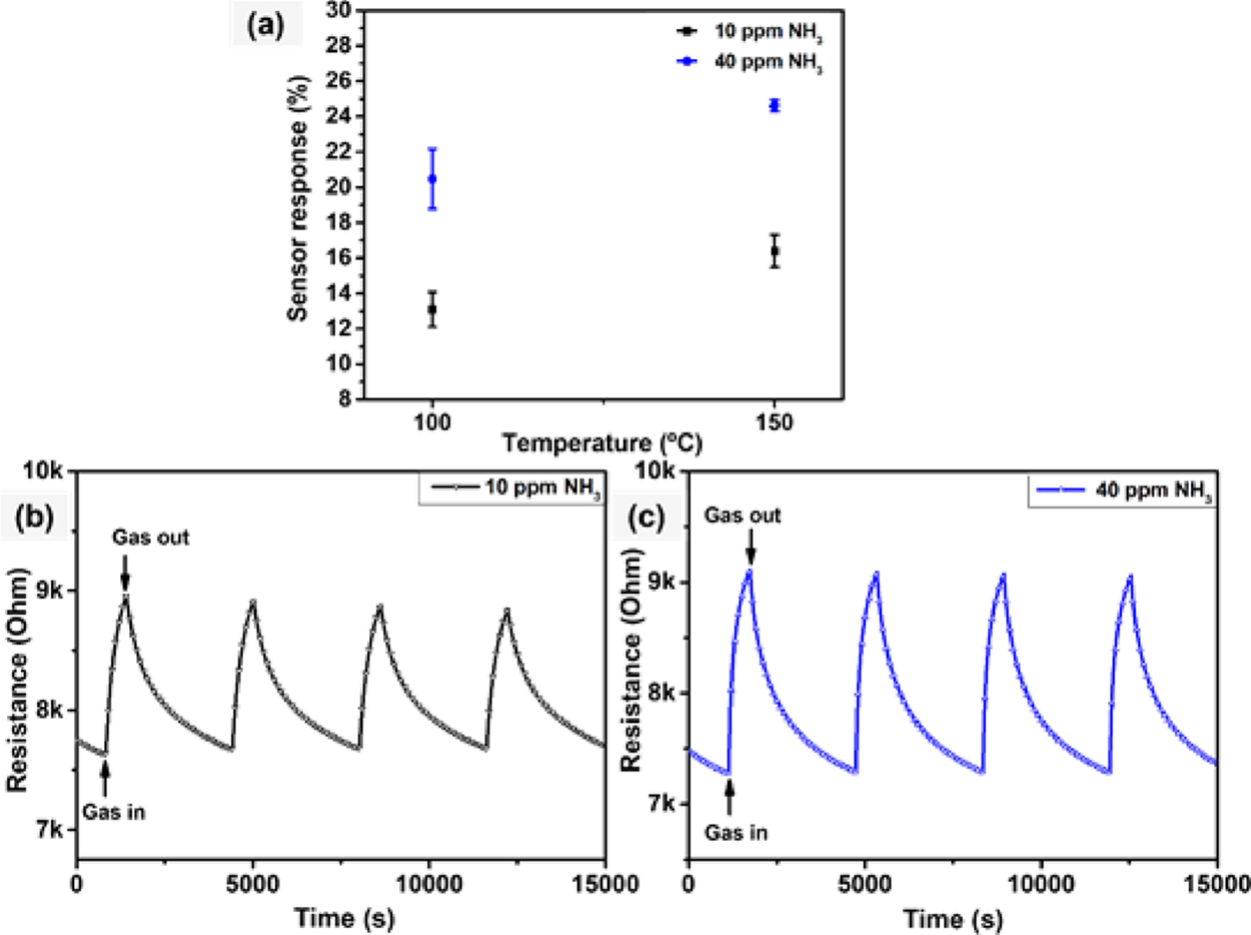
(a) WSe_2_ sensor response as a function of temperature toward NH_3_ gas, (b) film resistance change as a function of time toward (b) 10 ppm of NH_3_ and (c) 40 ppm of NH_3_, at 150 °C.

Aside from NO_2_ gas sensing, the performance of the WSe_2_ nanoflower sensors against NH_3_ gas was also investigated. Ammonia emissions are often related to livestock, food manufacturing, and textile industries. This species is of particular interest because of its adverse effects on human health and the environment at higher concentrations than NO_2_. Nevertheless, the exposure to even low levels of ammonia can irritate the nose and throat.^51^ We investigated the optimal working temperature for the WSe_2_ nanoflower sensors in the presence of NH_3_ vapors. As shown in Figure 9a, the sensor response increases linearly with temperature, with practically little response at room temperature (Figure S2), which can be expected due to rapid reaction rates at elevated temperatures. This behavior is more prominent when the concentration of the target gas is increased from 10 to 40 ppm. For instance, the gas response at 100 and 150 °C is 13 and 15.5%, respectively, toward 10 ppm NH_3_ gas. Furthermore, as the NH_3_ gas concentration is further increased to 40 ppm, the calculated response rises to values of 20.5 and 24.5% at 100 and 150 °C, respectively. This can be attributed to the hierarchical nanoflower structure, resulting in a high surface area which in turn enhances the active sites for NH_3_ adsorption and surface reactions. Similar results were reported for self-assembled 2D and 1D WS_2_ nanomaterials, which were synthesized by adopting a similar methodology, resulting in enhanced gas sensing properties toward NO_2_ gas detection. Moreover, DFT calculations indicate that the adsorption energies of NO_2_ and NH_3_ onto a WSe_2_ monolayer are −0.32 and −0.44 eV, respectively, thereby explaining that different optimal operating temperatures may apply for the two analytes tested.^52^ Therefore, considering the higher ammonia response recorded at 150 °C, this temperature has been considered for subsequent measurements with NH_3_ gas. Higher operating temperatures were not considered for preventing the thermal degradation (oxidation) of the gas-sensitive films.

Figure 9b,c shows typical repeated response and recovery cycles for WSe_2_ sensors toward 10 and 40 ppm of NH_3_, respectively. As mentioned earlier, these sensors show p-type semiconducting properties resulting in increased electrical resistance upon exposure to NH_3_ molecules (i.e., a reducing gas). Furthermore, sensors exhibit a repeatable response at each concentration, with the response and recovery time being substantially shorter for the higher NH_3_ concentration (40 ppm) (Figure S3). For instance, for 10 ppm of NH_3_, the WSe_2_ sensor has a response time of 460 s and a recovery time of 2282 s (Figure S3a). For 40 ppm, the sensor has a response time of 396 s and a recovery time of 1917 s (Figure S3b). When response and recovery dynamics are limited by the diffusion of gas molecules within the gas-sensitive film, response and recovery times decrease with gas concentration.^53,54^ The lengthy recovery time is associated to the strong interaction of NH_3_ molecules and the surface of the sensitive material. While such an interaction promotes high sensitivity for analyte detection, this is at the cost of suffering from difficult desorption of adsorbed ammonia molecules.

**Figure 10:**
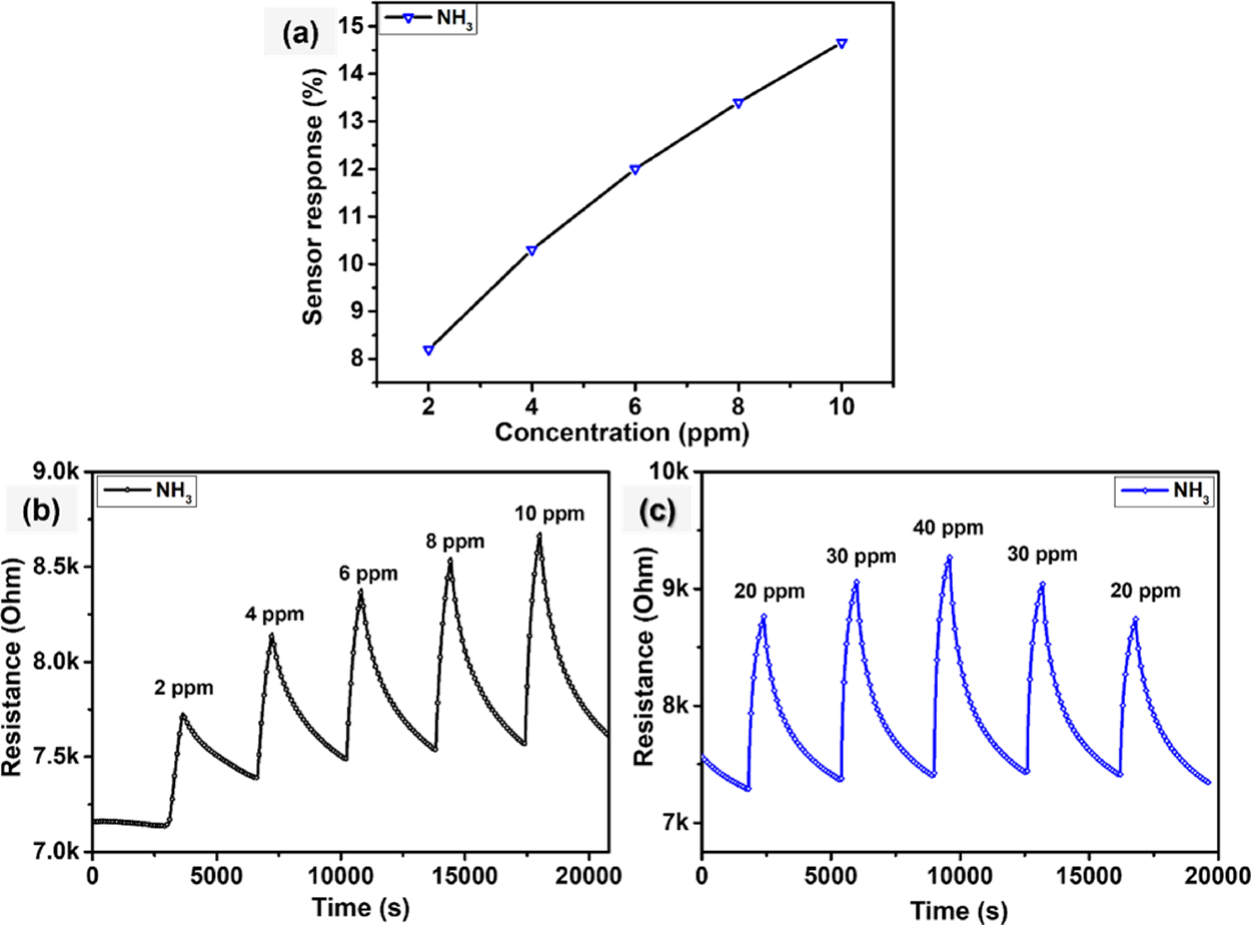
(a) WSe_2_ sensor response as a function of NH_3_ concentrations, (b,c) film resistance change as a function of time toward different concentrations of NH3 at 150 °C.

Figure 10a demonstrates the response to varied NH_3_ concentrations at a constant operating temperature of 150 °C. The results reveal that as the concentration of analyte increases, so does the sensing response. During this measurement, a sensor was exposed to five successive NH_3_ concentration pulses ranging from 2 to 10 ppm, as illustrated in Figure 10b. For 10 min, a diluted mixture of NH_3_ in dry air was injected at concentrations of 2, 4, 6, 8, and 10 ppm. The WSe_2_ sensor response was calculated to be 8.2, 10.3, 12, 13.4, and 14.5% toward 2, 4, 6, 8, and 10 ppm of NH_3_, respectively. In addition, as shown in Figure 9c, a sensor was evaluated for increasing and decreasing NH_3_ gas pulses from 20 to 40 ppm and vice versa. During this measurement, the sensor was exposed to NH_3_ pulses of 20, 30, 40, 30, and 20 ppm. As expected, the sensing response increases with increasing analyte concentration. For instance, the sensor response increased from 15.6 to 24.6% toward 10 and 40 ppm NH_3_, respectively. It is evident from the results obtained that the sensor shows reproducible and repeatable responses.

##### 3.1.6.3 Humidity

**Figure 11:**
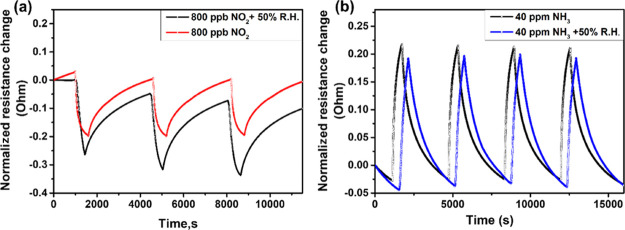
Dry and relative humidity cross-sensitivity to (a) 0.8 ppm NO_2_ at 100 °C and (b) 40 ppm NH_3_ at 150 °C.

To verify the practicability of the WSe_2_ sensor, the influence of relative humidity on the sensor response toward NO_2_ and NH_3_ gases was also investigated. The typical results are presented in Figure 11a,b, where sensors were tested at 100 °C for 0.8 ppm NO_2_ and 150 °C for 40 ppm NH_3_ in a 50% humidified air background. It was observed that the sensor response was increased from 20.5 to 26% and the baseline resistance was slightly decreased from 12 to 10 kΩ when the sensor was subjected to 0.8 ppm NO_2_ gas in the presence of ambient moisture (50% R.H). Similar behaviors regarding the moisture-enhanced NO_2_ response have previously been reported in the literature.^55^ In Figure 11, sensor resistance changes are calculated, as defined in eqs 1 and 2, when under dry conditions. Under humid conditions, *R*_air_ is replaced by *R*_air_50%RH and *R*_gas_ is replaced by *R*_gas_50%RH.

However, an extremely small change in sensor response as well as the baseline resistance is observed when exposed to NH_3_ diluted in humidified air. The sensor response to 40 ppm NH_3_ is calculated to be 24.1% in the presence of 50% RH, indicating an almost negligible change in its sensing response toward ammonia (response was 24.6% under dry conditions) and the baseline resistance was 7.4 kΩ in dry air and becomes 6.9 kΩ in the presence of 50% RH. This difference is in the range of the uncertainty associated to measurements. Moreover, water vapor is widely recognized for interfering with gas detection by changing the sensor electrical resistance in a similar way to a reducing gas. The great resilience of the sensor films, on the other hand, illustrates the WSe_2_ nanoflower sensor strong reproducibility and stability for detecting NH_3_ vapors, even under humid conditions.^55^ Also, it is worth mentioning that only very few studies,^21,24,47^ particularly for WSe_2_-based gas sensors, have reported their behavior under humid conditions. None of the previously reported results for WSe_2_ have reached such low humidity cross-sensitivity as we report here.

In this respect, when compared to prior research studies using WSe_2_ and other TMDs material, the WSe_2_ sensor fabricated in this work shows high performance (Table 2), with high sensitivity, low LoD, and outstanding (i.e., extremely low) humidity cross-sensitivity.

**Table 2:** Ammonia Gas Sensing Characteristics Reported in This Work Are Compared with Various TMDs Materials^a^

2D material	studied conc. (ppm)	working temp. (°C)	response	LoD (ppm)	selectivity study	response time	humidity cross-sensitivity test	ref
**WSe_2_ NFs**	**40**	**150**	**24.65% ± 0.31**	**2 ppm (exper.)**	**NO_2_, C_6_H_6_, CO and H_2_**	**396 s**	**negligible effect of humidity**	**this work**
3D WSe_2_ on 5 L Al_2_O_3_	50	150	1.3 (*R*_g_/*R*_a_)	not available	NO_2_, CO, C_3_H_6_O	not available	not studied	(63)
2D WSe_2_ on 5 L Al_2_O_3_	50	150	1.1 (*R*_g_/*R*_a_)	not available	NO_2_, CO, C_3_H_6_O	not available	not studied	(63)
MoS_2_ ns	10	100	30%	2 ppm (exper.)	CO and H_2_	193 s	not studied	(28)
WS_2_ aerogel	800	250	–0.8 (Δ*R*/*R*_0_)	13 ppm (theor.)	H_**2**_ and NO_**2**_	180 s	decrease in sensor response under wet conditions	(59)
Cu_2_S thin films	500	25	19.78%	not available	not available	60 s	not studied	(64)
SnS_2_	100	200	7.4%	0.5 ppm (exper.)	CO_2_, H_2_, CH_4_, ethanol, acetone	40.6 s	not studied	(65)
mixed-phase WS_2_	100	150	4.72	1 ppm (exper.)	C_3_H_8_O, C_6_H_5_Cl, C_6_H_12_O_2_, C_2_H_5_OH, C_7_H_8_,	19 s	N/A^b^	(66)
WS_2_ thin films	5	25	–0.019% (Δ*R*/*R*_0_)	1.2 ppm (theor.)	not available	not available	not studied	(67)

a NFs: nanoflowers; 5 L: 5 layers; ns: nanosheets; conc.: concentration exper.: experimentally verified; theor.: theoretically calculated.

b This study was conducted at a fixed low humidity level (28%) only and there are no data available to determine the effects of varying moisture levels on ammonia response.

##### 3.1.6.4 Selectivity

One of the most essential criteria in determining a sensor performance is selectivity. At 150 °C, we tested our sensors selectivity by exposing them to fixed concentrations of various species such as benzene vapors, carbon monoxide, and hydrogen. These gases or vapors are also of particular importance because they represent substantial health risks. For example, exposure to 5 ppm of benzene vapors for more than 15 min has been linked to the development of cancer.^68^

**Figure 12:**
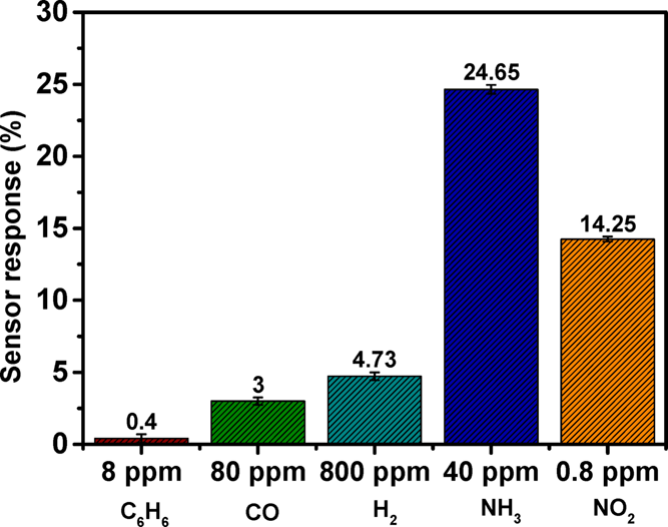
WSe_2_ nanoflower sensor response based on exposure to 8 ppm C_6_H_6_, 80 ppm CO, 800 ppm H_2_, 40 ppm NH_3_, and 0.8 ppm NO_2_ gas at an operating temperature of 150 °C.

The results in Figure 12 reveal the WSe_2_ nanoflower sensor selectivity for NH_3_ gas, with a maximum response of 24.65% and minimal responses to benzene and carbon monoxide. In addition to NH_3_, the sensor exhibits some small reactivity to hydrogen gas (5%). However, it must be noticed that the hydrogen concentration tested was 20 times higher than the NH_3_ concentration. The sensor also responds moderately (14%) to 0.8 ppm NO_2_ gas at 150 °C, which could be ascribed to the dual selective nature of the WSe_2_ sensor. Although the sensor shows dual selectivity toward NH_3_ and NO_2_ detection, it cannot be denied that the sensor is significantly more sensitive to NH_3_ than to NO_2_ at 150 °C. The ammonia and nitrogen dioxide concentration levels tested in this selectivity study have been selected close to the recommended exposure levels (REL) of the National Institute for Occupational Safety and Health (NIOSH). To address the current cross-sensitivity challenges and enhance the sensor selectivity toward NH_3_ gas detection, functionalization with different nanomaterials might be taken into consideration in this line of research. Furthermore, it is important to consider the sensor’s great resistance toward humidity cross-sensitivity. In this respect, these research findings could pave a way for the development of WSe_2_-based sensors that, in contrast to other TMDs-based gas sensors and many common metal oxide-based gas sensors, can operate at lower temperatures with ambient moisture.

**Figure 13:**
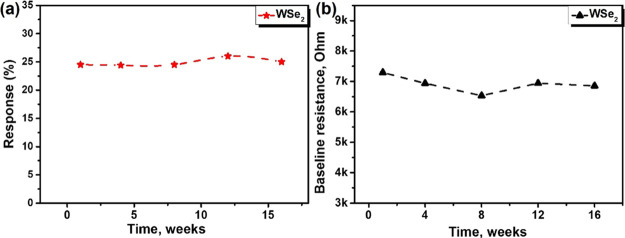
Long-term stability study. (a) Stability study for the WSe_2_ sensor toward NH_3_ gas over time, (b) evolution of the baseline resistance with time.

Additionally, the stability of the sensors has been examined and the outcomes are depicted in Figure 13. The evolution of sensor response and baseline resistance was studied when NH_3_ measurements (40 ppm) were repeated at regular intervals over a prolonged period (4 months). Even though some changes appear in the baseline resistance of WSe_2_ sensors during the period in which long-term stability was studied, sensors display an almost constant response toward ammonia (computed according to eq 2) over 4 months. Throughout the whole long-term stability study, sensors were stored in the laboratory under ambient humidity and temperature conditions.

### 3.2 Gas Sensing Mechanism

A gas sensor performance is linked to its sensing mechanism. While in traditional metal oxide-based sensors, their gas sensing mechanism is based on a surface reaction between the analyte gas molecules and pre-adsorbed oxygen ions on the metal-oxide surface. TMD-based gas sensors, on the other hand, depend completely on the adsorption/desorption and charge transfer processes between the target gas and the reactive sites in these materials.^67^

**Figure 14:**
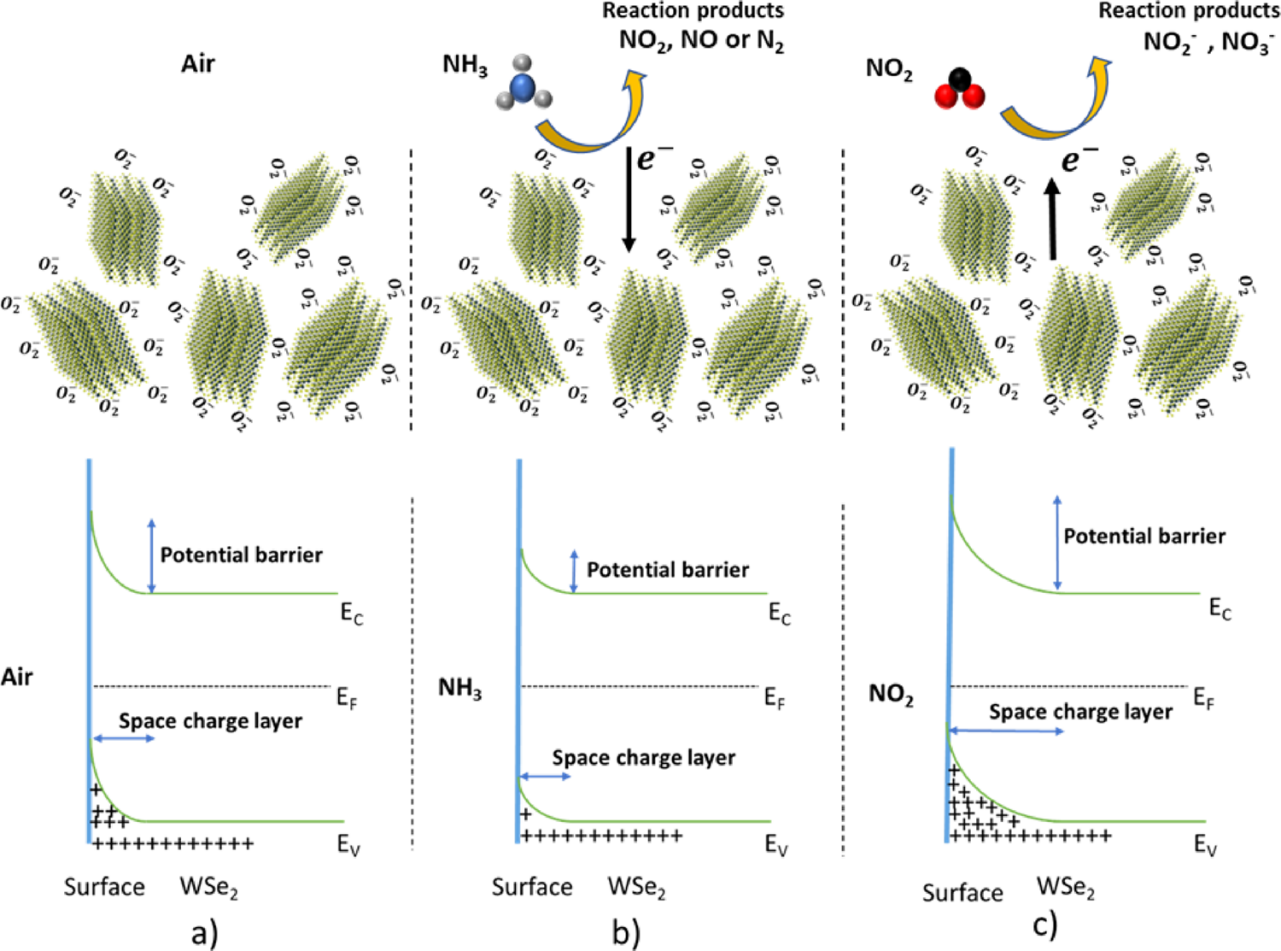
Schematic sketch of NH_3_ and NO_2_ gas sensing mechanisms and associated energy band diagrams for 2H WSe_2_ (a) in air, (b) in the presence of NH_3_, (c) in the presence of NO_2_, where *E*_C_, *E*_F_, and *E*_V_, correspond to the conduction band, Fermi level, and valence band.

The NH_3_ gas sensing mechanism of 2D WSe_2_ nanoflowers could be illustrated as the physisorption of the NH_3_ gas molecules and charge transfer between WSe_2_ nanoflowers and NH_3._^39^ As a result of the induced charge transfer, the conductance of the material changes. To better understand the sensing mechanism, a concise schematic is illustrated in Figure 14.

Meanwhile, to fully comprehend the gas sensing mechanism, the presence of oxygen in ambient air cannot be neglected, as it is one of the prominent absorbates during the gas sensing measurements.^47,69^ Therefore, the gas sensing mechanism of a 2H WSe_2_ is based on two consecutive reactions occurring at the WSe_2_ surface. Initially, when the sensor is exposed to dry synthetic air, oxygen molecules are adsorbed on the exposed surface.^47^ From the literature, it is well known that the adsorbed oxygen species exists as O_2_^®^ (<150 °C), O^®^ (150–400 °C), and O^2–^ (>400 °C).^12^ Because all the gas sensing measurements are performed at and below 150 °C (owing to the thermal degradation of WSe_2_ material at a temperature above 200 °C), the adsorbed oxygen molecule is equivalent to O_2_^®^. The as-adsorbed oxygen molecule results in the extraction of electrons from the valence band of WSe_2_ which results in the formation of a hole accumulation region (HAR) near the valence band, as shown in Figure 14a. The reaction can be represented by the following equation.
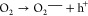
3

Afterward, when the sensor is exposed to NH_3_ gas, the spontaneously adsorbed nucleophilic molecules donate electrons to the WSe_2_ surface as well as they may react to the pre-adsorbed oxygen species present at the surface, resulting in the formation of free electrons. The free electrons that are released during the reaction combine with holes present in the valence band, thereby reducing the concentration of holes and subsequently increasing the resistivity of the sensor (as shown in Figure 8). As a result, the concentration of holes in the HAR region is reduced (Figure 14b). The p-type semiconductor behavior of WSe_2_ nanoflowers could be described by the following equation:^70^
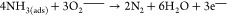
4

As the NH_3_ gas concentration is further increased, the electron density increases as well, improving the sensor sensitivity to rising gas concentrations and decreasing the response and recovery time (see Figures 10b,c and S3). Furthermore, when the gas is removed and the sensor is exposed only to dry air at room temperature, there is an inadequate recovery of the WSe_2_ sensor because of the difficulties in the desorption of surface species at room temperature. However, mild heating of the sensor at 150 °C resulted in complete baseline recovery. Therefore, 150 °C was chosen as the optimal working temperature toward NH_3_ detection.

Contrary to this, when the sensor is exposed to NO_2_ molecules, an opposite behavior is seen owing to the oxidizing nature of NO_2_ gas. Because NO_2_ molecules are electrophilic, they extract electrons from the valence band of 2D WSe_2_ nanoflowers, causing electron deficit and a rise in hole concentration (Figure 14c). Consequently, the electrical conductance of the material increases, thereby resulting in a decrease in sensor resistance, as shown in Figure 6 (where the baseline resistance of the WSe_2_ nanoflower decreases when exposed to NO_2_ gas). Similar to NH_3_, NO_2_ gas molecules might as well interact with the preabsorbed oxygen species at the WSe_2_ surface, resulting in the formation of NO_3_^®^. In addition to reaction 3, other reactions occurring at the surface of WSe_2_ can be described by the following equations:^47^
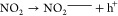
5
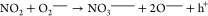
6

Furthermore, both edge atoms and in-plane defects of WSe_2_ have previously been demonstrated to be active sites for molecule adsorption, resulting in providing additional surface areas for gas adsorption, thereby enhancing the overall gas sensing. Moreover, these reactions are particularly prevalent at the 2H defect sites, mainly at the Se vacancies. These vacancies contribute to the carrier charge transfer on the TMD surface and play a crucial role in the gas sensing mechanism because prior research has shown that the adsorption of NO_2_ molecules is impossible without Se vacancies.^71^ This is further supported by DFT calculations, which demonstrated that in the absence of disulfide vacancies, the adsorption of N_2_ molecules on MoS_2_ is low.^72^ Despite the lack of research in the case of WSe_2_, current studies on MoS_2_ suggest that sulfur vacancies are one of the major defects in MoS_2_ because of their low formation energy.

Gas sensing might also benefit from the active sites on the edges of these 2D materials. The 2D morphology of the WSe_2_ nanoflowers that are attached to the 1D WSe_2_ nanowires plays a significant role in providing a high surface area for the adsorption of these gaseous molecules (NO_2_ and NH_3_). Similar reports in the literature have revealed enhanced gas sensing characteristics of vertically distributed MoS_2_ nanostructures supported by vertically arranged carbon nanotubes in comparison to horizontally arranged MoS_2_.^73^ Moreover, in our previous studies, we have reported enhanced gas sensing properties of 2D assemblies of WS_2_ nanoflowers on 1D nanowires in comparison to the more closely packed nanoflake assembly.^17^

Pristine TMD nanomaterials such as MoTe_2_, MoS_2_, and WS_2_ have been found to be sensitive to nitrogen dioxide and ammonia. In contrast, such materials show weak responses toward other species such as carbon monoxide, hydrogen, or aromatic volatile organic compounds (a-VOCs).^29,52^ Unlike NH_3_ and NO_2_, other species such as CO, H_2_, and a-VOCs too weakly interact with the surface and the edges of TMDs. This low interaction energy results in a weak charge transfer between the molecule and the TMD film, which translates into a small chemoresistive signal. Some authors have reported the doping (e.g., with phosphorous or palladium) of TMDs as a way to enhance their response to CO^74^ or H_2_,^75^ respectively. This could explain why our WSe_2_ sensors operated at 150 °C are more sensitive to ammonia than to any of the other reducing gases tested.

## 4 Conclusions

We used an atmospheric pressure CVD approach to demonstrate a simple, effective, and high-yield synthesis process to obtain WSe_2_ films. The morphology of as-grown WSe_2_ is nanoflowers, which are composed of highly crystalline vertically aligned nanoplatelets. Furthermore, the synthesis approach is scalable and enables the direct growth of nanostructured material over functional substrates. The as-grown material is used to develop a chemoresistive type gas sensor having dual sensitivity toward NH_3_ and NO_2_ gas detection, depending on the operating temperature used. To the best of our knowledge, no previous research on WSe_2_-based gas sensors has yielded such intriguing results. Moreover, the cross-sensitivity test revealed that H_2_, C_6_H_6_, and CO have a negligible effect on NH_3_ gas detection while the presence of NO_2_ molecules shows some cross-sensitivity which could be assigned to the dual sensitive nature of the WSe_2_ films. Moreover, water vapor at 50% relative humidity also resulted in having no interference to the measure of NH_3_ gas, attesting to promising characteristics of WSe_2_ for gas sensing applications. Henceforth, we believe that the results obtained in this study at moderate temperatures are intriguing and can provide a useful conceptual framework for detecting low concentrations of NH_3_ in a real environment.

## Supporting Information Available

Response and recovery times of WSe_2_ toward 800 ppb NO_2_; WSe_2_ sensor response toward 10 ppm NH_3_ gas at room temperature; and response and recovery time of the WSe_2_ sensor toward (a) 10 ppm, (b) 40 ppm, NH3 at 150 °C (PDF)
