# Supplementary material for: Three-Dimensional Assemblies of Edge-Enriched WSe2 Nanoflowers for Selectively Detecting Ammonia or Nitrogen Dioxide
Source: ACS Appl Mater Interfaces. 2022 Dec 5;14(49):54946–60. doi: 10.1021/acsami.2c16299 (PMC9756288; doi:10.1021/acsami.2c16299)
Supplement: Supplementary file 1 — am2c16299_si_001.pdf [file am2c16299_si_001.pdf]

Supporting Information

3D assemblies of edge enriched WSe_2 nanoflowers for selectively detecting ammonia or nitrogen dioxide

Aanchal Alagh[a], Fatima Ezahra Annanouch*[a], Ayrton Sierra-Castillo[b], Emile Haye[c]

Jean-François Colomer*[b], Eduard Llobet*[a]

[a]: Department d'Enginyeria Electronica, Universitat Rovira I Virgili, Avenida Paisos Catalans 26,43007, Tarragona, Spain.

[b]: Laboratoire de Physique du solide (LPS), Namur Institute of Structured Matter (NISM), University of Namur, Rue de Bruxelles, 61, 500 Namur, Belgium.

[c]: Laboratoire d'Analyse par Réactions Nucléaires (LARN), Namur Insitute of Structured Matter (NISM), Université de Namur, Rue de Bruxelles 61, 5000 Namur, Belgium.

*Corresponding authors e-mails: fatimaezahra.annanouch@urv.cat, eduard.llobet@urv.cat; jean-francois.colomer@unamur.be

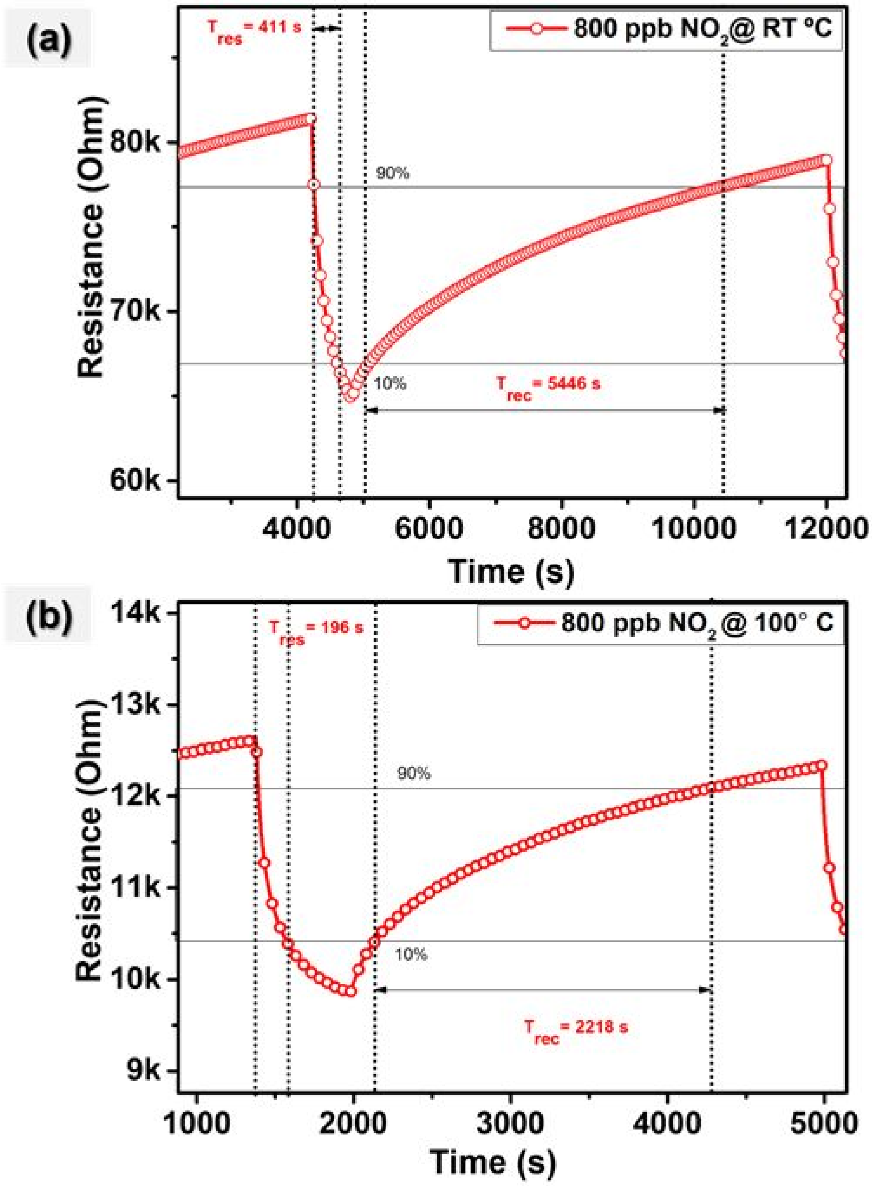

Figure S 1 Response and recovery time of WSe_2 towards 800 ppb NO_2 at (a) room temperature, (b) at 100 ºC.

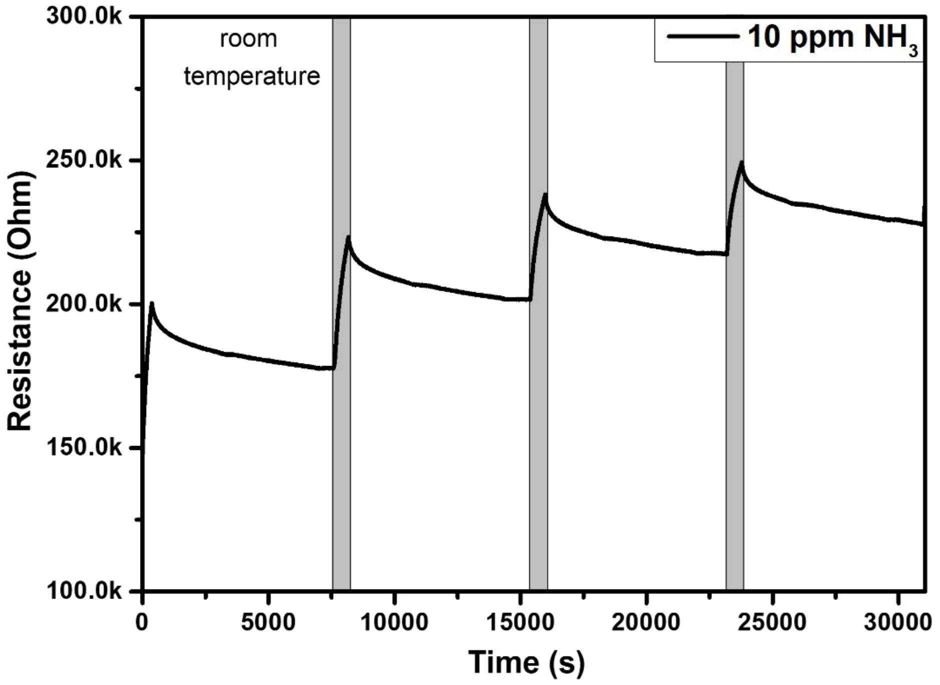

Figure S 2 WSe_2 sensor response towards 10 ppm NH_3 gas at room temperature.

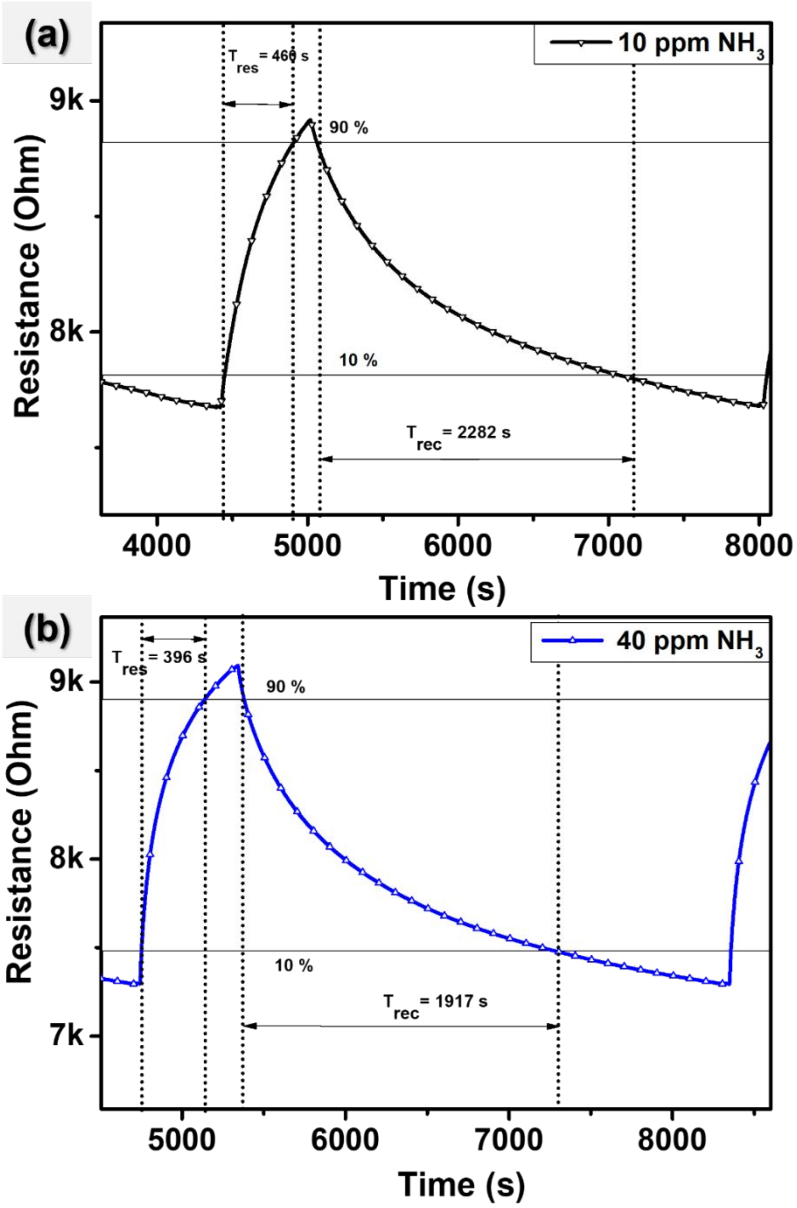

Figure S3 Response and recovery time of WSe2 sensor towards (a) 10 ppm, (b) 40 ppm, NH_3 at 150 ºC.
